# SinicView: A visualization environment for comparisons of multiple nucleotide sequence alignment tools

**DOI:** 10.1186/1471-2105-7-103

**Published:** 2006-03-02

**Authors:** Arthur Chun-Chieh Shih, DT Lee, Laurent Lin, Chin-Lin Peng, Shiang-Heng Chen, Yu-Wei Wu, Chun-Yi Wong, Meng-Yuan Chou, Tze-Chang Shiao, Mu-Fen Hsieh

**Affiliations:** 1 Institute of Information Science, Academia Sinica, Taipei, 115, Taiwan; 2 Genomics Research Center, Academia Sinica, Taipei, 115, Taiwan

## Abstract

**Background:**

Deluged by the rate and complexity of completed genomic sequences, the need to align longer sequences becomes more urgent, and many more tools have thus been developed. In the initial stage of genomic sequence analysis, a biologist is usually faced with the questions of how to choose the best tool to align sequences of interest and how to analyze and visualize the alignment results, and then with the question of whether poorly aligned regions produced by the tool are indeed not homologous or are just results due to inappropriate alignment tools or scoring systems used. Although several systematic evaluations of multiple sequence alignment (MSA) programs have been proposed, they may not provide a standard-bearer for most biologists because those poorly aligned regions in these evaluations are never discussed. Thus, a tool that allows cross comparison of the alignment results obtained by different tools simultaneously could help a biologist evaluate their correctness and accuracy.

**Results:**

In this paper, we present a versatile alignment visualization system, called SinicView, (for Sequence-aligning INnovative and Interactive Comparison VIEWer), which allows the user to efficiently compare and evaluate assorted nucleotide alignment results obtained by different tools. SinicView calculates similarity of the alignment outputs under a fixed window using the sum-of-pairs method and provides scoring profiles of each set of aligned sequences. The user can visually compare alignment results either in graphic scoring profiles or in plain text format of the aligned nucleotides along with the annotations information. We illustrate the capabilities of our visualization system by comparing alignment results obtained by MLAGAN, MAVID, and MULTIZ, respectively.

**Conclusion:**

With SinicView, users can use their own data sequences to compare various alignment tools or scoring systems and select the most suitable one to perform alignment in the initial stage of sequence analysis.

## Background

With exponentially increasing genomic sequences available in the public domain [1-5] comparative genomics demonstrates its power to help biologists identify novel conserved and functional regions in genomes [6-9]. Based on the comparison of cross-species genomic sequences, biologists can understand the evolutionary relationship of genomic regions among species, discover conserved regions between different genomes, such as yeast species genomes [10], metazoan genomes [11], vertebrate genomes [12], and mammalian genomes [13], discover regulatory motifs in the yeast [14] and human promoters [15] or identify potential conserved non-genic sequences (CNGs) [16].

However, genomic sequences can be megabase long and thus the traditional sequence alignment tools based on dynamic programming would not work efficiently due to their time and space complexities. To better tackle this problem, several tools for genomic sequence alignment have been proposed, such as pairwise sequence aligners like MUMmer [17], GS-Aligner [18], Avid [19] and LAGAN [20], and multiple sequence alignment (MSA) programs like T-COFFEE[21], MAFFT [22], MultiPipMaker [23], MULTIZ [24], MLAGAN [20], MAVID [25], and MUSCLE [26,27]. These alignment tools, however, are heuristics based and do not provide any indication of how far they are from an optimal solution. The comparisons of alignment tools using a set of benchmarking sequences have also been conducted in recent years [28-30]. We found that the majority of these tools usually fail to generate consistent results especially in aligning divergent cross-species sequences. As a result, the more alignment tools there are available in the public domain, the more confusion it creates for users to decide which tool is most suitable to align their sequences.

Although the comparison results in [28-31] provide some evaluations of several popular alignment tools, the conclusions may not be directly applicable to users' sequences. Furthermore the user usually does not know for sure whether those poorly aligned regions produced by the alignment tools are indeed non-homologous or just due to inappropriate tools or scoring systems used. Consequently, if some homologous regions are unaligned, the estimated evolution distances of these sequences may be inaccurate and therefore the constructed phylogenetic trees may be incorrect. Facing this problem, the user may have to try different tools or scoring systems to evaluate the correctness and accuracy of alignment results in the initial stage of sequence analysis. On the other hand, new alignment tools are released continually. Users may want to compare these newly released tools with those that they are most familiar with. Thus, it is desirable and most useful to have a visualization system that provides a *direct *and efficient method and can assist users to cross compare and inspect alignment results obtained by different MSA tools especially at the initial stage of sequence analysis.

In recent years, a number of visualization tools have been released in the public domain. These tools can be roughly divided into two categories: integrated genome/sequence browser and individual alignment result visualization. In the former category, such as UCSC ENCODE project [32,33], UCSC human genome browser [34], *Ensembl *[35], ECR Browser [36,37], users can view alignment results mapped onto the sequenced genomes. Some of these browsers also provide registered users to submit alignment results and see the conservation regions between different genomes. In the latter category, the tools are developed to visualize individual alignment results. The VISTA-related tools are among the famous ones that have been developed for several years [38]. mVISTA is a set of programs for comparing DNA sequences from two or more species up to megabases long and visualize these alignments with annotation information [39]. rVISTA (regulatory Vista) combines database searches for transcription factor binding sites with a comparative sequence analysis [40,41]. GenomeVISTA compares users' sequences with several whole genome assemblies [42,43]. Phylo-VISTA analyzes alignments of multiple DNA sequences from different species while considering their phylogenetic relationships [44]. In general, the VISTA family of tools provides users with a novel graphical user interface (GUI) to view alignment results from different viewpoints. In addition to the VISTA family, PipMaker [23,45], and zPicture [46] are also popular visualization tools for sequence or genomes alignment results. All of these tools are web-based with friendly user interfaces, and allow users to easily visualize alignment results with annotations. However, these tools are limited solely to single alignment results. The capability of simultaneously comparing multiple results from different alignment tools or different parameters of a scoring system, such as changing match rewards or mismatch penalties, is notably lacking.

In this article, we present a versatile alignment visualization system, SinicView (Sequence-aligning INnovative and Interactive Comparison VIEWer), which enables users to efficiently compare and evaluate assorted alignment results obtained by different tools. SinicView for the present calculates similarity of the alignment outputs under a fixed window using the sum-of-pairs method and provides scoring profiles of each set of aligned sequences. Other scoring matrices, such as EMBOSS DNA scoring matrix [47] and YASS [48], are also provided in SinicView for users to select. Besides, users can also upload their preferable scoring matrices to calculate the scoring profile curves. Users can visually compare alignment results either in graphic scoring profiles or in plain text format of the aligned nucleotides. In addition, the information about alignment gaps and sequence annotations is also presented. The real-time juxtaposition of the visualization results from different MSA programs would bring more insights into the evaluation process. With SinicView, users can use their own sequences to survey and compare various multiple alignment tools and thus to unveil their merits (and shortcomings). Moreover, the cross-tools comparison can provide users more confidence in their final alignment results especially for those poorly aligned regions.

## Implementation

There are three viewing sections in SinicView: Global View, Detailed View, and Information View (including annotations and gaps.) The Global View section shows the whole percent identity plots that calculate the sum-of-pair scores based on one specified reference sequence. In the Detailed View section, the panels show the whole percent identity plots of different alignment results individually. By observing the graphical results, it is much more intuitive and straightforward to judge the consistency of the alignment results. When the sliding window is less than 100 base pairs, the Detailed View section will automatically switch from the curve-based plot to the display of the detailed alignments in a colored text format where identical characters are shown. The Information View section containing annotation and gap information is stacked beneath the Detailed View section. SinicView also provides several global comparison charts that can assist biologists to choose the best alignment result among those produced by the programs under consideration. SinicView is implemented entirely in Java language to ensure portability across major platforms and is accessible with a web browser and Internet connection. The main features of SinicView are summarized as follows:

1. Visualization of the scoring distribution of alignment results in a curve-based graphic format;

2. Generation of the comparison charts using stacked-bar and pie charts, which shows the distribution of the identical rates among various alignment programs for benchmarking purposes;

3. Inclusion of a versatile manipulative functionality (gap-display toggling, drag-and-drop zooming/shifting, and graphic/text display toggling);

4. Visualization of annotation information and display of the phylogenetic trees provided by users in which the drawing tree program uses the ATVtree [49];

5. Visualization of detailed text alignments results;

6. Capability to export the visualization results to portable image files.

In what follows, we will introduce the characteristics and functionality of SinicView in more detail.

### Manipulative operations in SinicView

**Figure 1:**
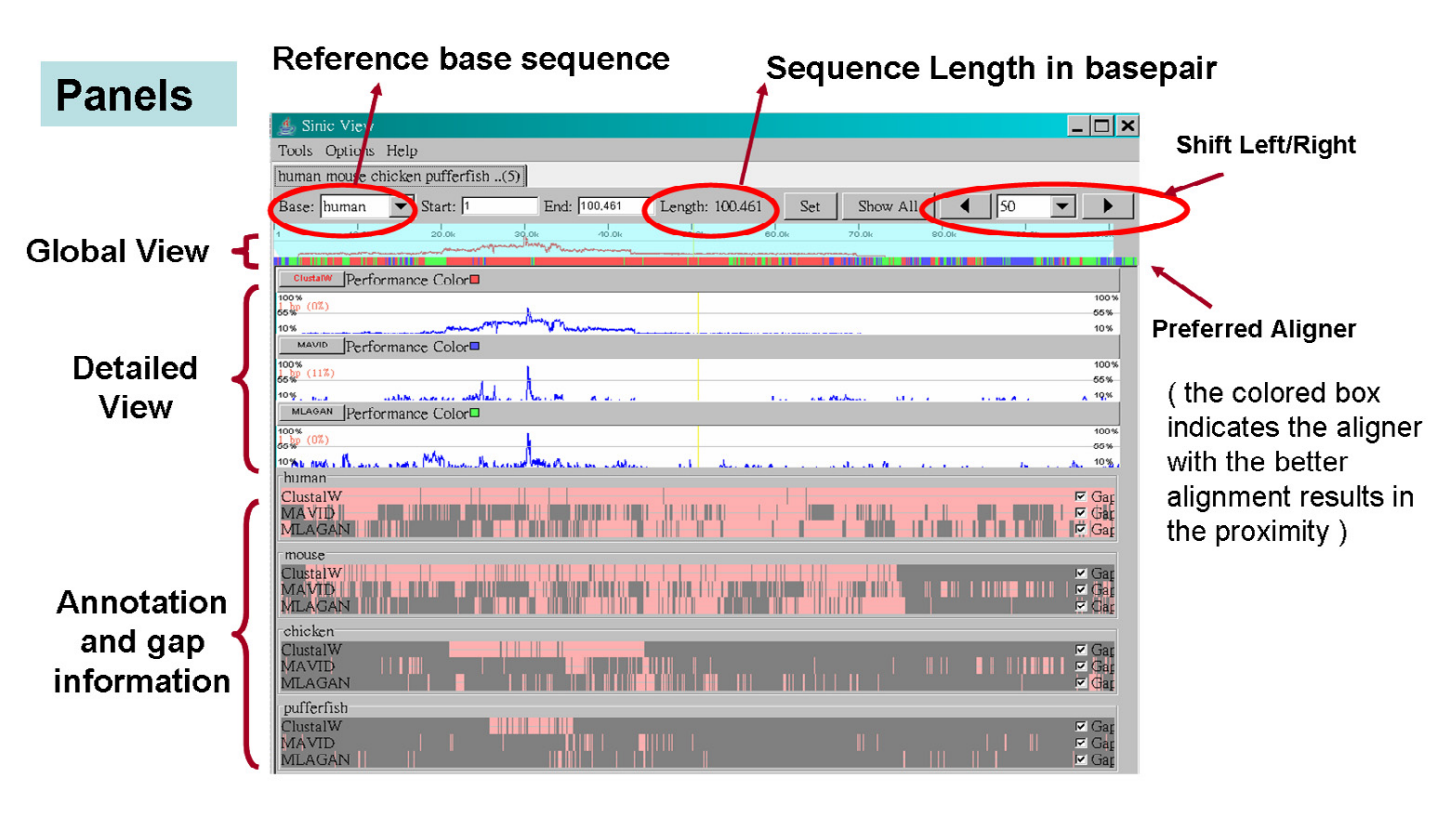
**The screenshot shows the user interface of SinicView. **The alignment result is of the SCL gene regions in human, mouse, chicken, pufferfish, and zebrafish. Three alignment results of five sequences aligned by ClustalW, MAVID, and MLAGAN are shown.

SinicView offers a series of manipulative and navigational controls, such as zooming, shifting, and gap/annotation toggling. As shown in Figure 1, SinicView displays the alignment results obtained by three different MSA methods. The input sequences contain the orthologous regions around the Stem Cell Leukemia (SCL) gene in five vertebrate species: human, mouse, chicken, pufferfish and zebrafish. The buttons and text-field boxes of manipulative functions are located on top of the frame. Users can manually input numerical values or click on the highlighted colored region in the Global View section that specifies the zooming or shifting factors in a drag-and-drop fashion. When the highlighted region is clicked and dragged, the equivalent of a shift action will be performed and the display region can be resized by adjusting the edge of the highlighted area.

SinicView can display more than one alignment result obtained by different alignment programs (either pairwise or multiple ones.) The assorted mixed-color span under the Global View panel shows among the alignment tools used the preferred aligner, which generates comparatively better results on the spot. Each of the aligners is denoted by a pre-defined color with the "performance color" label right next to the name of the tool.

### Multi-panel functionality in SinicView

In the Detailed View section, the Percent Identity Plot (PIP) panels show, from top to bottom, the similarity curves of the alignment results obtained by different programs, along with the names of the alignment tools. In the Information View section, the Gap & Annotation panels (in pink and gray) display the information of annotations provided by users, and gaps of aligned sequences. The information and similarity ratios can also be displayed as the current scan-line (i.e. cursor) moves. The boxes in maroon denote the annotation area and the horizontal line represents the original sequences interleaved with inserted gaps (light gray areas.) The gap display can be toggled on or off via the checkbox on the right.

Because different alignment results are usually of different lengths, it is not plausible to compare these results base-pair by base-pair. In SinicView, therefore, we let users select one of input sequences as a *reference *and then calculate the sum-of-pair scores of each base pair in the reference within a fixed window. For example, each alignment result in the PIP panels at the scan-line position corresponds to human sequence, selected as the reference in Figure 1. When the user selects different sequences as the reference, SinicView can demonstrate the variations between the PIP curves of the alignment results.

### Visualization of SinicView: comparison chart and text-mode comparison

**Figure 2:**
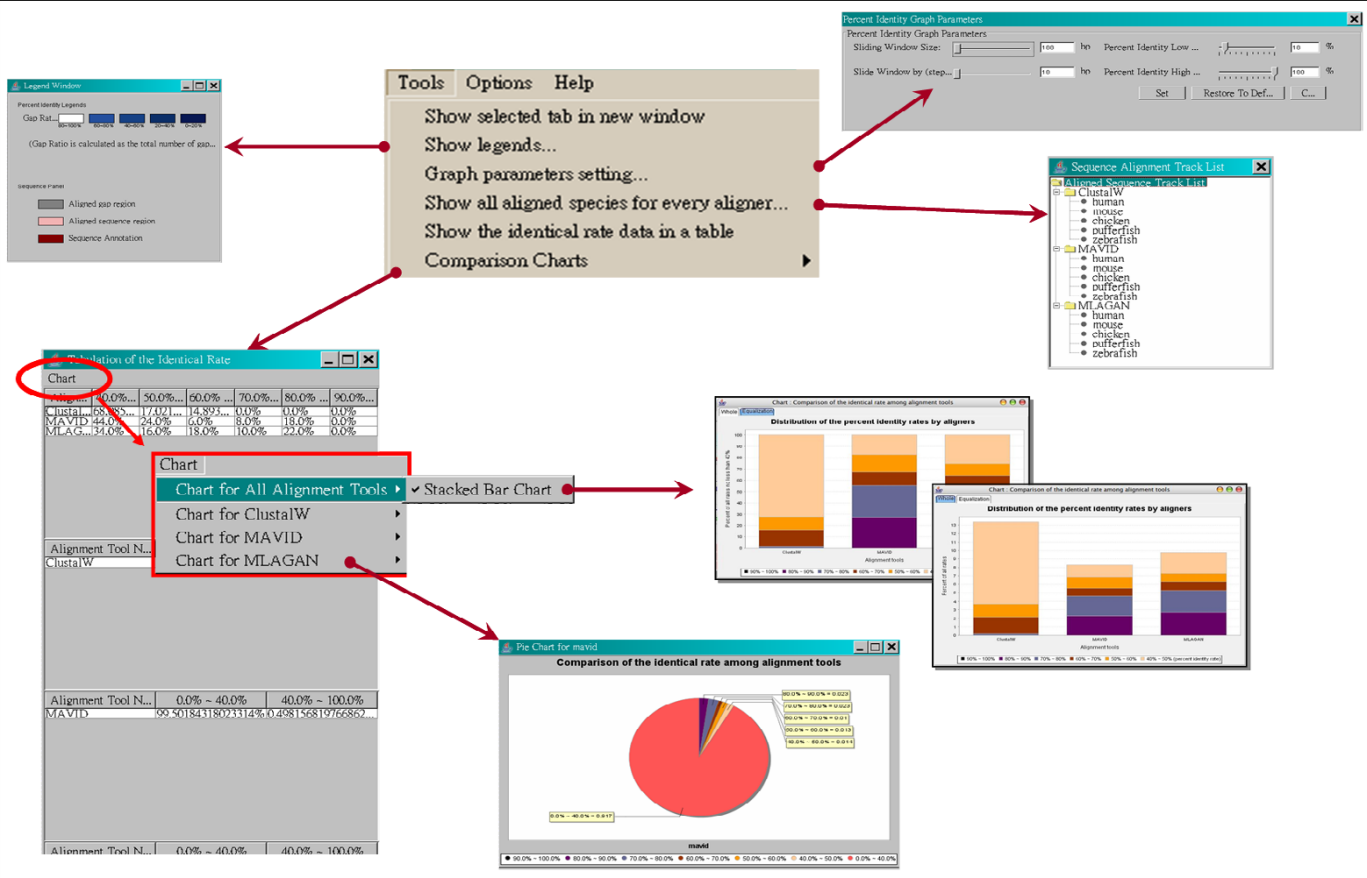
**The tools menu functions. **Two comparison charts can be generated by SinicView: the stacked-bar chart illustrates the proportion comparison of cross alignment results and the pie chart shows the proportion of different identical rates of an individual alignment result. The complete data of the charts are tabulated on the left.

The functionality under the "Tools" menu, called "Comparison Charts", offers two types of charts for quick-and-easy evaluation of the alignment quality. The stacked bar chart, in Figure 2, illustrates the distribution of the identical rates with the threshold over 40%. The pie chart, on the other hand, displays the distribution of the identical rates from 0 to 100 percent based upon a selected alignment program. The statistics on which these charts are based can also be displayed in a tabulated text form.

**Figure 3:**
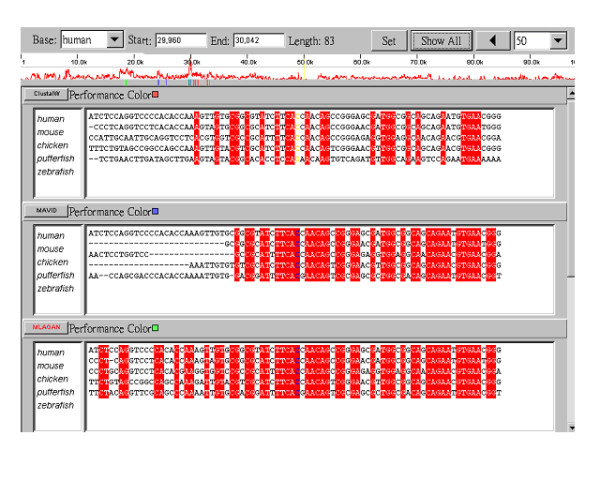
**The detailed text display of the different alignment results. **The matched identical sequences are labeled in red blocks. Interestingly, all three results do not contain consistent matching alignments in this case.

SinicView also provides a plain-text view of the alignment results in the Detailed View section when the sliding window size is less than 100 aligned base pairs. As shown in Figure 3, the plain-text alignment results replace the percent identity curves and the fully identical bases in a column are labeled in red blocks. Thus, users can check the correctness of detailed alignment results base pair by base pair.

### Installation and execution of the standalone SinicView

The applet version can be accessed via any JRE (Java Runtime Environment)-enabled browsers with Internet connection, thus making the installation and choosing the right platform hassle-free. However, the ease of running SinicView on-the-go cannot accommodate the bandwidth requirement in case of huge amount of sequence data involved. Hence, we have also implemented a standalone application of SinicView, which is wrapped in JRE, for off-line use.

The execution procedure of the standalone SinicView is quite straightforward. Upon launch, the user will be prompted three options. The first two are to read user's Phylogenetic Tree files, an option, and MSA results from the local disk.

## Results

In what follows, we will introduce two examples to demonstrate how SinicView can assist users to analyze alignment results in the initial stage of sequence comparison. The total alignment lengths in both of the examples are few hundreds of thousands of base pairs and several millions of base pairs, respectively. The conservations of the aligned sequences are different in each example. More examples can be found in [50].

### Example 1: SCL (Stem Cell Leukemia) gene

The Stem Cell Leukemia (SCL) gene plays a critical role in normal processes that, when disrupted, can result in leukemia. The *SCL *gene, also known as *tal-1*, encodes a basic helix-loop-helix transcription factor that is pivotal for the normal development of all hematopoietic lineages, and is highly conserved between mammals and zebrafish [51,52]. Previous analyses of the SCL genes in five vertebrate genomes, including human, mouse, chicken, pufferfish, and zebrafish, have revealed that the SCL promoter/enhancer motifs are conserved in all five species [51]. The alignment and visualization tools used in their analyses included BLAST [53], PipMaker [45], and DiAlign [54]. Shah et al. (2004) realigned these gene regions in five species by a pairwise alignment tool, LAGAN [20], and demonstrated the alignment result by Phylo-VISTA [44]. In this paper, we also downloaded these sequences and realigned them by the multiple alignment tools: ClustalW, MAVID and MLAGAN. The lengths of the human, mouse, chicken, pufferfish, and zebrafish sequences are approximately 100 kb, 65 kb, 67 kb, 22 kb, and 8 kb, respectively.

**Figure 4:**
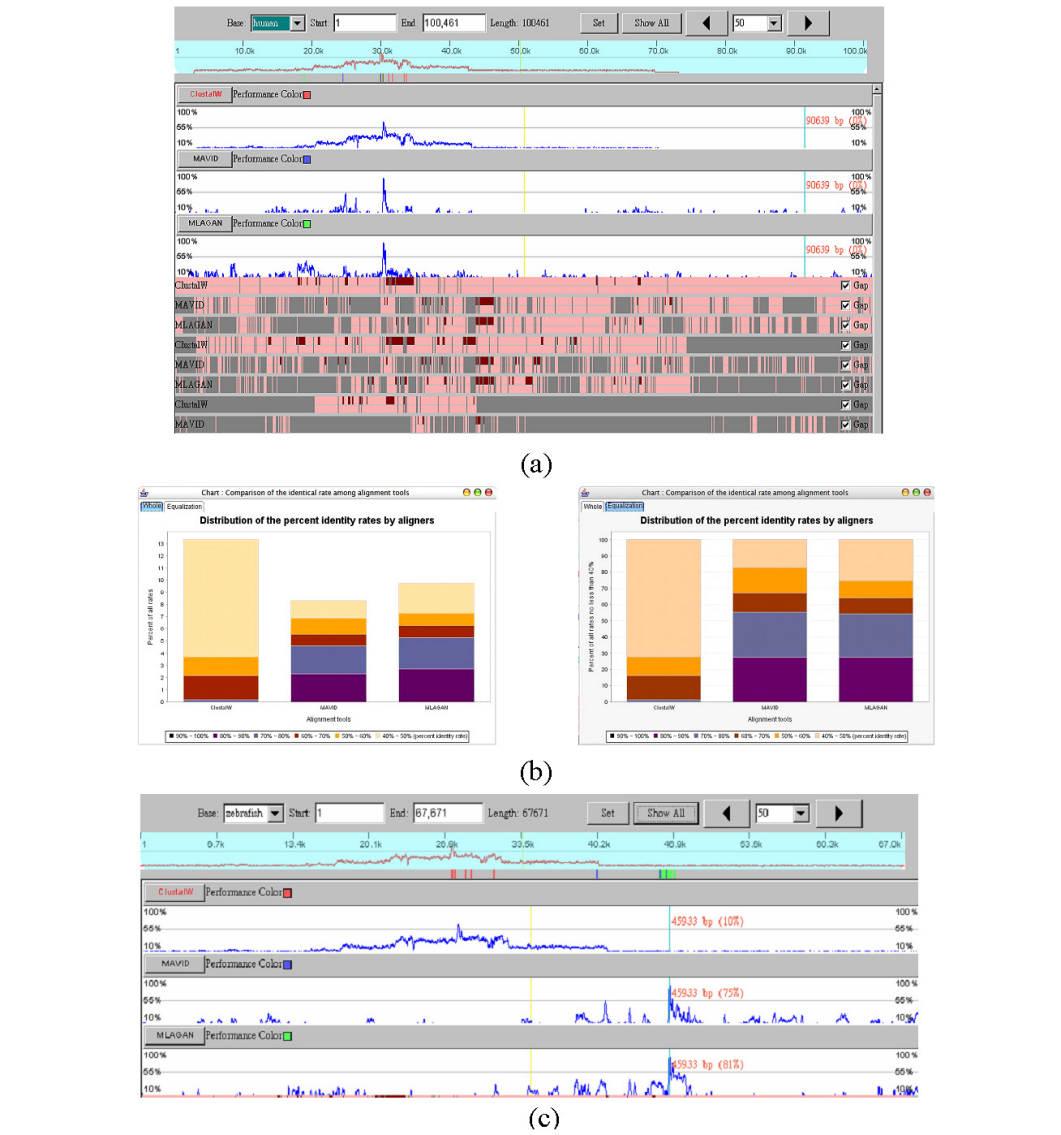
**The comparison of different alignment results of SCL gene regions. **(a) The comparison of three alignment results by SinicView while using the human sequence as the reference. (b) The whole (non-equalization) and equalization stacked-bar charts generated by SinicView illustrates the proportion comparison of cross alignment results. (c) Using zebrafish as the reference, the highest conserved region (around 62%) produced by ClustalW concentrates around at 27.5 k bp. However, there are discrepancies between the result of ClustalW and those of MAVID and MLAGAN.

Figure 4(a) shows the global view of the results obtained by three alignment tools using the human sequence as the reference. Generally speaking, the highest conserved region located at 30 k bp of human sequence is all well aligned by these three tools. But the highest identical rates of the alignment by ClustalW are lower than those by either MLAGAN or MAVID. Moreover, the total quantity of the result obtained by MLAGAN is better than those by both ClustalW and MAVID while the quantity of the result obtained by ClustalW is better than those by the others, as shown in Figure 4(b). Interestingly, when we selected the zebrafish sequence as the reference, the result obtained by ClustalW shows the highest conserved region located at around 27.5 k bp whereas those by both MAVID and MLAGAN show it at around 45.89 k bp, as shown in Figure 4(c). The comparison reveals that the region at around 27.5 k bp in the zebrafish sequence will be assumed the homologous region by ClustalW. But according to MAVID and MLAGAN, the homologous regions are located at around 45.89 k bp rather than at 27.5 k bp. This ambiguous result may be caused by segmental duplication in the sequences and by difference in alignment strategy. In this case, more advanced or further inspections should be performed to either check the detailed alignment results in both regions or realign these sequences by using other pairwise or local alignment tools.

### Example 2: The greater CFTR region

The cystic fibrosis transmembrane conductance regulator (CFTR) gene is responsible for the cystic fibrosis disorder that spans approximately 190 k bp of genomic DNA and consists of 27 exons [55]. The greater CFTR region is defined as a genomic segment of about 1.8 M bp on human chromosome 7q31.3 containing the CFTR gene and nine other genes, including TES1, CAV1, CAV2, MET, CAPZA2, ST7, WNT2, GASZ, and CORTBP2 [12]. The comparative analysis of this region in 13 vertebrate species has been reported in Thomas et al., 2003 [12] in which the alignment tool used was BlastZ on PipMaker Web server [45]. In this paper, we downloaded the sequences of four mammalian species, including human, baboon, dog, and mouse, from the NIH Intramural Sequencing Center (NISC) Website [56]. However, the original sequences had been updated in other genome browsers. Thus, we eventually downloaded the last versions of these sequences from the UCSC Genome Browser. The lengths of these sequences are from 1.0 M bp to 1.5 M bp. We realigned these sequences by MLAGAN, MAVID, and TBA (kernel: MULTIZ) [24] and the total number of bases of the final alignment results, including gaps, are approximately 12 M bp, 11 M bp, and 7.5 M bp, respectively.

**Figure 5:**
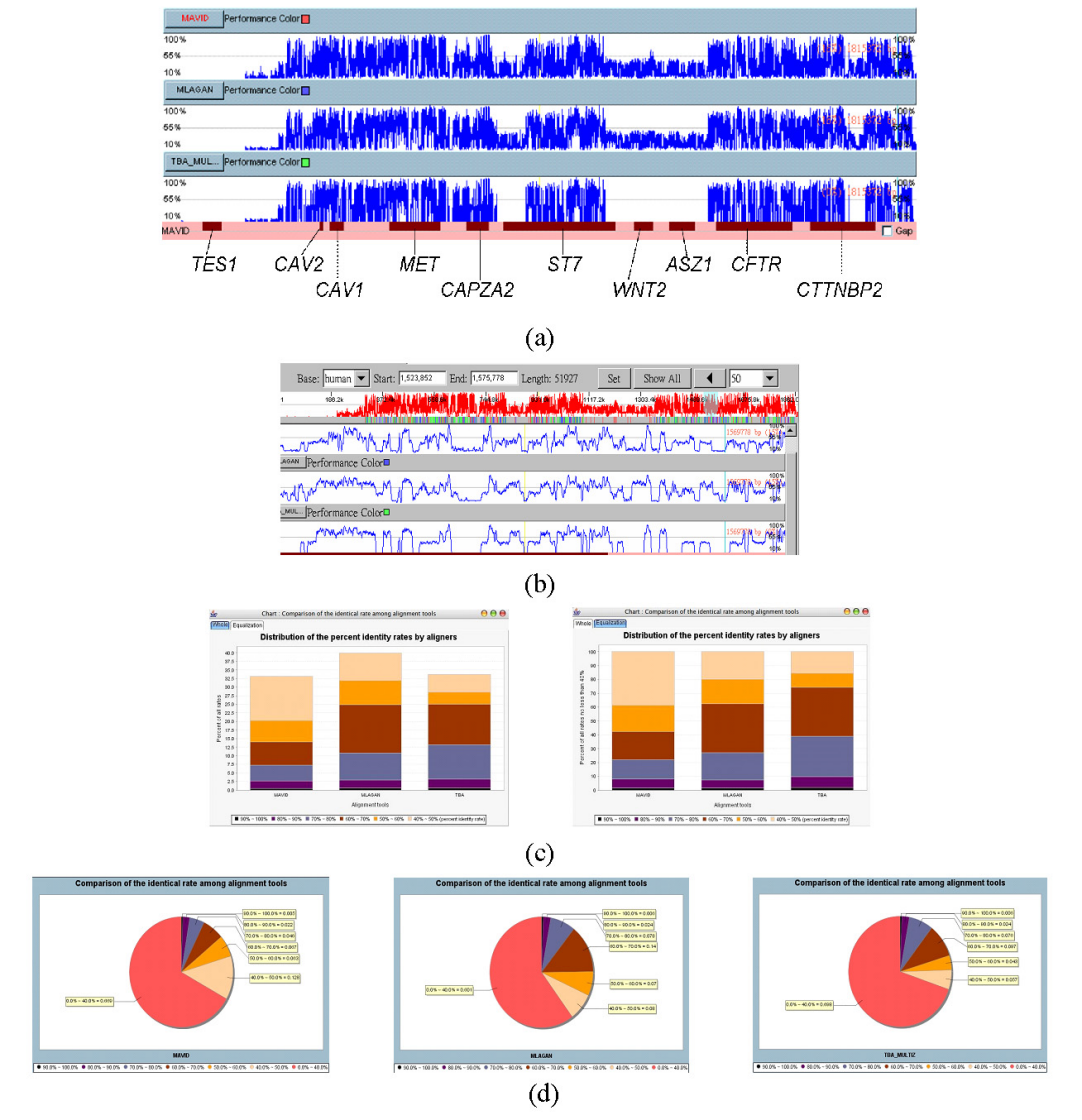
**The comparison of different alignment results of great CFTR gene regions. **The cross comparison of three alignment results by SinicView. (a) The whole scale PIP curves using the human one as reference. (b) The detailed view of (a). (c) Comparison of the results in the whole and equalization stacked-bar charts. (d) Comparison of the results in the pie charts.

Figures 5(a) and 5(b) show the global PIP curves and their detailed views of three alignment results, respectively. In general, most of high identity regions are well and consistently aligned by these three programs. But those not as high identities are not reported by TBA because the kernel of this program, MULTIZ, is based on the local alignment results by BlastZ. As shown in Figure 5(c), the stacked-bar charts show the quality and the quantity of these alignment results where the average identical rates for TBA are somewhat better than those for MLAGAN and MAVID although the total number of aligned conserved regions for MLAGAN is larger than those for the others.

For comparisons of these alignments from a functional viewpoint, we downloaded the annotation of the human sequence, including exons and repeats, from the Ensembl Genome Browser [35]. The detailed comparisons of the alignment results by different aligners demonstrated that the alignments of noncoding regions are often inconsistent. But for the coding regions, the alignment results by different aligners seem consistent and well-aligned.

**Figure 6:**
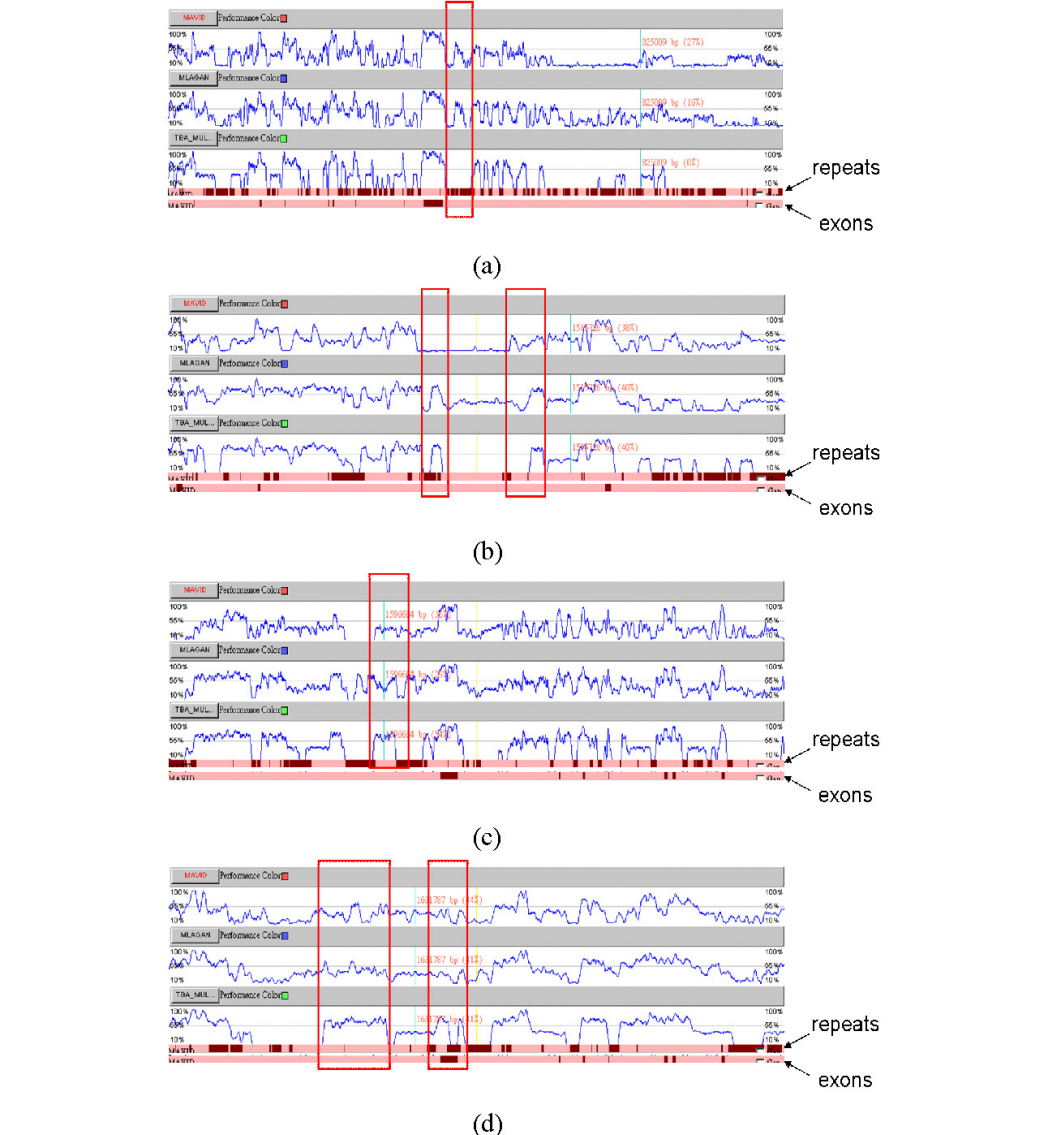
**The detailed comparison of Example 2. **The detailed comparison of different alignment results of great CFTR gene regions at different intervals. (a) From 786,112 bp to 836,774 bp. (b) From 1,500,792 bp to 1,523,689 bp. (c) From 1,583,342 bp to 1,621,404 bp. (d) From 1,623,603 bp to 1,644,063 bp.

Figures 6(a)–(b) show the detailed alignment results at four different intervals. In Figure 6(a), we find that some conserved regions are not aligned by TBA but identified by MLAGAN and MAVID. This region is annotated by repeats and implies that some repetitive elements were inserted into these sequences of their common ancestor. However, this conserved insertion event could not be observed by using TBA. Although the kernel of TBA, MULTIZ, is known not to align regions with repetitive elements, we still find that some other regions with repetitive elements are aligned by this program, as shown in Figure 6(b).

Generally speaking, the regions aligned by TBA usually have higher identical rates than by others. As the frames shown in red in Figures 6(c) and 6(d), the alignment of these regions by TBA seems superior to those by others. However, the kernel of TBA, MULTIZ, usually neglects to align the regions with low conservations. Thus, some lowly conserved regions may not be aligned by TBA.

Since each alignment tool has its own advantage and reveals different alignment results, we therefore wonder whether a better alignment result can be generated by hybridization of these alignment tools.

### Loading performance and platforms test

SinicView is implemented totally in Java. Theoretically, it should be portable across different operating systems (OSs) and platforms. To demonstrate interoperability on real cases, we tested the applet and application versions of SinicView on different platforms and OSs. As shown in Table 1, both versions of SinicView seem to perform well. Thus, users can use either the applet version or the standalone application of SinicView, according to their requirements.

Besides, we also tested the loading performance of SinicView. Because the performance of an applet on the Web is strongly dependent on the network bandwidth and traffic, the estimation of loading time may not be a fair comparison. Thus, in this part we only estimated the loading performance of the standalone application of SinicView.

In general, the loading performance of a Java application is dependent on the memory heap size. The default values of the initial heap size and the maximum size of a Java Virtual Machine (java_1.4.2 version or higher) are 4 M (mega) bytes and 64 M bytes, respectively. These values can be adjusted by the following command in the terminal mode:

java -Xms64m -Xmx128m -jar SinicView.jar,

where the parameters Xms64m and Xmx128m represent that the initial heap size is 64 M bytes and the maximum size is 128 M bytes, respectively. Thus, we used different input data sizes, initial heap sizes, and the maximum sizes to estimate the loading time of SinicView. As shown in Table 2, using the default maximum heap size, 64 M bytes, the standalone SinicView can handle up to approximately 11 M bytes alignment data. If the maximum size is set up to 256 M bytes, the loading ability of input data size could be over several dozens of mega bytes. Moreover, Table 2 shows that the maximum data size is dependent on the maximum heap size and the loading times are linearly dependent on the sizes of input data. All performance test results were benchmarked on a 3 GHz Pentium4 PC with 1 GB RAM.

## Discussion

### Repetitive elements in sequence alignments

The eukaryotic genome is usually characterized by the presence of repetitive DNA consisting of nucleotide sequences of various lengths and compositions that occur from a few times to thousands of times in the genome either in tandem or in a dispersed fashion[57]. The repetitive fractions can be classified into two types of repeated families: localized and dispersed [57,58]. Localized repetitive sequences usually occur as tandem arrays and they are called tandem repetitive DNA. Dispersed repetitive sequences are dispersed throughout the genome. In addition, there are moderately repetitive sequences, which are usually transposable elements or processed pseudogenes and are usually dispersed over the genome. *Alu *is the largest family of interspersed mobile elements (~300 bp) and propagated to more than one million copies in primate genomes. This type of repeat has been inserted into these genomes within the last 65 million year period [58]. Because this type of repetitive elements only appears in the primate genomes, when we align homologous sequences of primate and non-primate genomic sequences, these *Alu *inserted regions should not be aligned. However, other interspersed elements may possibly have been inserted into the ancestral sequence of mammalians. The regions of these repeats may be able to align together between the sequences of different mammalians, as shown in Example 2. However, these regions in the alignment results by different aligners are inconsistent. Since these repetitive elements in sequences could be detected by RepeatMasker [59], the poorly aligned regions may have to be checked whether they belong to repetitive elements.

### Comparative approach for alignment validity

As the comparison results using SinicView show, the alignments of sequences using different MSA tools are inconsistent. We begin to wonder whether the computational results obtained by different tools may in fact lead to different findings. For identification of alignment correlation, a need for additional checks of alignment validity by using different tools and scoring systems has been recognized in the literature [60]. Thus, a cross comparison approach along with visualization could provide an efficient and easy way for general users to verify and validate the alignment results as to whether the aligned regions are reasonable and whether those poorly aligned regions are indeed non-homologous.

### How to decide on a "good" alignment result

Except evaluation of the alignment quality by comparison charts in SinicView, how to decide on a good alignment with biological meanings may need much more experiences and knowledge. Sometimes, this judgment depends also on what kind of the biological problems users want to study. Here, we suggest some general rules for users to judge the alignments by biological meanings.

In the coding regions, a triplet of adjacent nucleotides constitutes a codon. Usually, the first two nucleotides are identical between the two sequences and allow the third one to be either identical or different. Thus, when the partial alignment results reveal the two-out-of-three regularity for each triplet, it may imply that the aligned regions are potential coding regions. This alignment result should be more biologically meaningful than those without the two-out-of-three regularity.

From molecular evolutionary viewpoint, nature prefers inserting or deleting considerable consecutive nucleotides together to interspersed individual nucleotides [57]. Thus, an alignment with consecutive gaps would be better than those with interspersed gaps.

If one of the alignment sequences has been annotated, the information is definitely useful for users to judge the alignment results by different aligners.

### Comparative environment to promote new alignment tools

It is not easy to promote newly developed tools because users usually cannot directly compare the new tools with the traditional ones. With SinicView, users can compare the alignment results obtained by different tools and select an appropriate one for further analysis. Thus, if the new tool can align more regions than those by the old ones and can also indicate their statistical significances, it will be welcomed and better received by the community. We would like to make SinicView available to the community of computational biologists. In addition to helping the user find a most appropriate alignment tool to use, SinicView may also be used to check whether previously obtained alignment results by different tools are worth a re-investigation, and see if this revisit of alignment results would lead to different conclusions.

### Further possible enhancements for SinicView

The capability of fine-tuning parameters relevant to the alignment process will be made available in a user-friendly interface. Furthermore, the ability to allow plug-ins of more alignment programs, in addition to the currently pre-selected ones, such as ClustalW, MAVID, MLAGAN, and GS-Aligner, will inevitably broaden the usage of SinicView. The issue of the compatibility of the input and output formats for each alignment tool also needs to be resolved. For example, both MAVID and MLAGAN require the phylogenetic tree data as input, but ClustalW does not. The ordering of the outputs of these aforementioned tools is usually switched without notice. Thus, to be able to work under a unified comparison framework requires further processing of these outputs. Besides, identifying a standard-bearer mechanism is still a challenge in entrusting existing alignment programs. So far, we have used the "sum-of-pairs" method to define the "identical rate" in each alignment result. In the future, we may provide other criteria for users to use to measure their alignment results, in addition to what have been already provided in SinicView.

## Conclusion

Deluged by the increasing number of completed genomic sequences, biologists have encountered a challenge of aligning more and much longer sequences from divergent species. Thus, the need to align longer sequences, like mega base-pair sequences or even genome-scale sequences, and evaluate the alignment results becomes more urgent. In this paper, we have presented a visualization tool for comparison of multiple sequence alignment programs. With a standard simple protocol for the input/output format, it is quite easy for users to upload their own alignment programs to SinicView. The performance of SinicView depends on the system's internal memory. In a 64 M RAM JAVA environment, SinicView can load and visualize several mega bases alignment results. Users can easily perform sequence alignment by employing multiple alignment tools and visualize the results on the fly by SinicView. More information can be found at [50].

## Availability and requirements

Project name: 1. Development of Novel Large-scale Sequence Alignment and Visualization Tools and Their Applications to Bioinformatics

2. Development of a web-based personalized research environment for study of computational and evolutionary genomics

Project home page: 

Operating system(s): Window XP, Sun OS 5.7 Sparc, Mac OS 10.4.2 Tiger, and Linux Fedora Core 3

Programming language: Java

Other requirements: Java 1.4.2 or higher

License: Any restrictions to use by non-academics: free downloads and usage for academics only.

## List of abbreviations

**SinicView**: Sequence-aligning INnovative and Interactive Comparison VIEWer

**JRE**: Java Runtime Environment

**SCL**: Stem Cell Leukemia

**CFTR**: Cystic Fibrosis Transmembrane Conductance Regulator

## Authors' contributions

Arthur Chun-Chieh Shih and D.T. Lee contributed the original idea, developed the system organization, and drafted the paper. Laurent Lin supervised the system implementation and also drafted some parts of the paper. Chin-Lin Peng, Yu-Wei Wu, Chun-Yi Wong, Meng-Yuan Chou, and Tze-Chang Shiao implemented the codes. Shiang-Heng Chen and Mu-Fen Hsieh implemented some partial codes before leaving their positions.

## Figures and Tables

**Table 1:** The test results of the applet version and standalone application of SinicView on different platforms and OS's

	Applet		Standalone Application	
	
	Specification (Applet)	Status	Specification (Application)	Status
Sun OS	OS : Sun OS 5.7 Sparc JVM : java_1.4.2_08	**OK**	OS : Sun OS 5.7 Sparc JVM : java_1.4.2_08	**OK**
Mac OS	OS : Mac OS 10.4.2 Tiger JVM : java_1.4.2_08 java_1.5 update 4 Browser : Safari 2.0	**OK**	OS : Mac OS Tiger 10.4.2 java_1.5 update 4 java_1.5 update 4	**OK**
Linux/Unix	OS : Linux Fedora Core 3 JVM : java_1.4.2_08 Browser : Mozilla Firefox 1.0.2	**OK**	OS : Linux Fedora Core 3 JVM : java_1.4.2_08	**OK**
Windows	OS : Windows XP Service Pack 2 JVM : java_1.4.2_08 java_1.5 update 4 Browser : Internet Explorer 6.0 Mozilla Firefox 1.0.4	**OK**	OS : Windows XP Service Pack 2 JVM : java_1.4.2_08 java_1.5 update 4	**OK**

**Table 2:** The loading performance of standalone SinicView The loading time of standalone SinicView by different sizes of input data and initial and maximum memory heap sizes. The default value for the initial JVM heap size is 4 M bytes; maximum is 64 M bytes. For the maximum 64 M byte heap size, the standalone SinicView can handle up to approximately 11 M byte alignment data. The maximum value of the input data size is linear in the maximum heap size. We observe that the initial heap memory size has little impact on the loading time. This result was benchmarked on a 3 GHz Pentium4 PC with 1 GB RAM.

Input data size (bytes)	Loading Time (sec) Java Application Virtual Machine Memory Heap Size, Initial/Max (M Bytes)				
	
	64 MB/64 MB	128 MB/128 MB	64 MB/256 MB	128 Mb/256 MB	256 MB/256 MB
0.5 M	4	4	4	4	4
1 M	6	7	8	8	7
5 M	28	27	27	26	26
10 M	59	53	56	55	55
20 M	NA	104	107	105	106
40 M	NA	NA	214	212	212

NA: Not available.
